# Patient-centered goal-setting in stroke rehabilitation: a scoping review

**DOI:** 10.3389/fresc.2026.1744900

**Published:** 2026-03-17

**Authors:** Inge Ris, Mille Nabsen Marwaa, Rikke Westengaard Nielsen, Palle Larsen, Hanne Kaae Kristensen

**Affiliations:** 1 Health Sciences Research Centre, University College Lillebaelt, Odense, Denmark; 2 Department of Sports Science and Clinical Biomechanics, University of Southern Denmark, Odense, Denmark; 3 Department of Physiotherapy Education, University College Southern Denmark, Esbjerg, Denmark; 4 Centre for Innovative Medical Technology, Department of Clinical Research, University of Southern Denmark, Odense, Denmark

**Keywords:** cross-sectional study, goal-setting, patient-centered, scoping review, stroke, stroke rehabilitation

## Abstract

**Background:**

Patient-centered goal-setting is an important part of the rehabilitation process. The guidelines for stroke rehabilitation in adults recommend setting goals that are meaningful and relevant for the patient, focusing on activity and participation, and involving the patient. Patient-centered goal-setting is to improve rehabilitation outcomes. However, patient-centered goal-setting occurs partly or not at all. There is also a lack of continuity in goal-setting across sectors.

**Objective:**

This study aimed to identify existing research-based knowledge on procedures used in patient-centered goal-setting processes in stroke rehabilitation.

**Methods:**

A scoping review was conducted by searching PubMed, CINAHL Complete, EMBASE, APA PsycINFO, Scopus, and Cochrane databases for studies involving adults receiving or clinicians delivering stroke rehabilitation and focusing on patient-centered goal-setting processes. The included studies were analyzed using inductive content analyses and linked to five domains in goal-setting processes: person-centeredness, collaboration with healthcare professionals and patients, coordination across sectors, monitoring, and evaluation.

**Results:**

Eighteen studies were included. Inductive content analysis identified elements related to goal-setting processes, mainly occurring at the beginning of the rehabilitation. Linking the studies to five domains revealed gaps in cross-sectoral coordination, monitoring, and evaluation.

**Conclusion:**

Patient-centered goal-setting in stroke rehabilitation is practiced variably, and there is no overall agreement about the procedures to ensure that goal-setting is patient-centered. Therapist- and team-led goal-setting processes are used. Evaluation procedures and cross-sectoral coordination are rarely described.

## 1 Introduction

In Europe, the annual incidence of stroke ranges from 95 to 290 per 100,000 individuals, and approximately 1.12 million Europeans suffer a stroke yearly despite progress in pharmacological interventions. This number is expected to increase by 27% in the European Union by 2047 due to the aging population and higher stroke survival rates (1, 2). In Denmark, approximately 170,000 individuals are living with the long-term effects of a stroke and almost 20,000 individuals suffer a brain hemorrhage, blood clot, or transient ischemic attack yearly, with 25%–30% requiring some form of rehabilitation (3).

After an acute stroke, patients should ideally be offered early interdisciplinary rehabilitation in a specialized stroke unit in a hospital and continue the rehabilitation process at a specialized neuro-rehabilitation and/or municipal rehabilitation unit. The rehabilitation should ideally be task-specific, three hours daily, five days weekly. Eventually, at a later stage, long-lasting, ongoing rehabilitation can take place in primary care clinics (4–6). For example, in 2022, in the Region of Southern Denmark, 63% of hospitalized stroke survivors were referred to primary care, with primary care physiotherapy costs of almost 16 million Euros (7, 8).

The Danish Health Authorities recommend that cross-sectoral stroke rehabilitation is patient-centered, based on the patient's overall functional capacity, needs, resources, potential, and goals. Health professionals in regions, municipalities, and primary care clinics should actively involve and collaborate with patients, their relatives, and relevant professionals when planning the rehabilitation pathway based on the patient's goals (9–11). In addition, each sector should have a goal-setting process, which can vary depending on the sector and rehabilitation phase. However, there is a lack of continuity across sectors in goal-setting, regarding patient-centeredness and goal-setting tools (12, 13). Moreover, patients want continuity and involvement in the transition between sectors (13). When this is lacking, patients are reported to perceive transitions between sectors as uncertain, experience anxiety, and potentially have unrealistic expectations (4, 14).

Patient-centered goal-setting is an important part of the rehabilitation process. The “National Institute for Health and Care Excellence—NICE” guidelines for stroke rehabilitation in adults recommend setting goals that are meaningful and relevant for the patient, focusing on activity and participation, and involving the patient (5). They state that goals should be set within the first five days and reviewed regularly with the patient. Goal-setting can be described as a formal process in which a rehabilitation professional or team, together with the patient and/or their family, make a collective decision following an informed discussion of how and when to conduct rehabilitation interventions (15). When a patient's problems require the involvement of two or more individuals from different professions and/or the process continues for more than a few days, a formal goal-setting process may be needed to motivate the patient, ensure that individual team members work towards the same goals, ensure that important actions are not overlooked, and allow monitoring of changes to end ineffective interventions (16).

Patient-centeredness, from a patient perspective, includes three domains: communication, partnership, and health promotion (17). Person-centeredness is also used in the context of rehabilitation. There is a large overlap between patient- and person centeredness (18). Person-centeredness in goal-setting is described as a process of interaction containing the search for a mutual understanding of what is meaningful to the patient and interaction skills (19).

Goal-setting appears to improve recovery, performance, and goal achievement and positively influences patients' perceptions of their self-care ability and engagement in rehabilitation and lack of clear goals influence the rehabilitation process negative (20, 21). However, two reviews found that patient-centered goal-setting was uncommon in acute stroke rehabilitation. Despite recommendations that patient-centered goal-setting should be standard practice and indications that patients could and want to be involved, goal-setting tends to be more clinician-, system-, or population-centered or does not occur (22, 23). In addition, in community hospitals, patient-centered goal-setting occurs partly or not at all, despite the contribution of goal-setting to patients' rehabilitation experience (23).

Mismatches between staff and patients' perspectives of goals and recovery, lack of expertise (by patients, their families, and staff), patients' impairments, lack of time and team cohesion, lack of clinical guidelines, lack of patient preparation, and high productivity expectations can complicate goal-setting in stroke rehabilitation (24). Conversely, tailoring the goals to patients' needs and preferences, providing support material, education (for staff, patients, and their families), appropriate resourcing, goal-setting guidelines, revised clinical routines, performance monitoring, and adequate time and resources can facilitate goal-setting (24, 25). Organizational factors such as resources, lack of supporting organizational processes, and factors related to health professionals' competence, knowledge, and skills also influence the implementation of patient-centered goal-setting in stroke rehabilitation (26).

In summary, there is a need for an updated overview of research-based knowledge of factors affecting patient-centered goal-setting in and across sectors of stroke rehabilitation to improve patient-centered stroke rehabilitation processes. Therefore, this scoping review aimed to identify and report existing research-based knowledge of patient-centered goal-setting in stroke rehabilitation.

## 2 Material and methods

### 2.1 Study design

A scoping review is considered the most appropriate methodology to identify and map existing research-based knowledge when the extent and nature of the research are largely unknown (27). This scoping review followed the JBI methodology for scoping reviews (28). The study protocol was published on the OSF Open Science Framework in July 2023 (https://osf.io/c79b5). This study is reported according to the Preferred Reporting Items for Systematic Reviews and Meta-Analyses (PRISMA) guidelines for scoping reviews (29).

### 2.2 Definitions

This study used the following definitions. Rehabilitation goal: A desired future state to be achieved by an individual with a disability through rehabilitation activities (30). Goal-setting processes: The establishment or negotiation of rehabilitation goals, which aim to facilitate the patient's autonomy, motivate the patient, ensure the team focuses on the same goals, ensure important actions are not overlooked, and ensure the monitoring of changes to end ineffective interventions (16, 30).

### 2.3 Participants and focus

The target population for this scoping review was adult stroke survivors (*patients*) and healthcare professionals (*clinicians*) involved in subacute and long-term stroke rehabilitation in public and private clinics. The clinicians were those who delivered stroke rehabilitation. This scoping review did not explore stroke rehabilitation goal-setting in the acute phase, as due to accelerated hospital stays in Denmark, almost 70% of patients are discharged after 10 days, and hardly any rehabilitation occurs in acute wards (3). Studies were included if goal-setting processes and/or activities were described as perceived by the patient and/or the clinicians delivering the rehabilitation.

### 2.4 Types of sources

This scoping review included studies with the following designs: (1) experimental and quasi-experimental studies, such as randomized and non-randomized controlled trials, before-after studies, and time-series studies; (2) analytical observational studies, such as prospective and retrospective cohort studies, case-control studies, and cross-sectional studies; (3) descriptive observational studies, such as case series, individual case reports, and descriptive cross-sectional studies; and (4) qualitative studies. Finally, we searched in the references of systematic reviews and clinical guidelines to identify relevant studies not identified in the systematic literature searches.

### 2.5 Search strategy

A preliminary search was conducted to identify relevant articles on the topic. Keywords and words in titles and abstracts were used as the basis of our full search. We were interested in three sets of search terms related to stroke, goal-setting, and rehabilitation. Two authors (IR and RWN) systematically searched PubMed, CINAHL Complete, EMBASE, APA PsycINFO, Scopus, and Cochrane databases to identify relevant articles published between June 2010 and September 2023. To our knowledge Rosewilliam et al. published the last review on this topic, so we limited our search to articles published since June 2010 (15). We updated our database search on 1st of July 2025 to identify relevant new studies.

### 2.6 Data selection

All identified articles were collated and uploaded into EndNote X9.3.3 (Clarivate Analytics, Philadelphia, PA, USA). Duplicates were deleted, and the articles were screened for eligibility based on the following five criteria: (1) Studies conducted in Western high-income countries (Australia, New Zealand, those in Northern America, and in Western Europe, or comparable countries); (2) Articles published in English, Dutch, Danish, Swedish, or Norwegian; (3) Articles reporting studies using any design except conference abstracts or reviews; (4) Studies involving adults receiving or clinicians delivering stroke rehabilitation except for those on acute stroke rehabilitation or speech therapy focusing on aphasia rehabilitation; and (5) Studies focusing on patient-centered goal-setting, and activities related to the establishment or negotiation of rehabilitation goals. All reviewers screened five randomly selected articles to discuss inclusion and exclusion before screening all articles for inclusion. Then, two reviewers (IR and MNW) independently screened all articles/citations for inclusion or exclusion. Disagreements regarding inclusion were resolved by consensus discussion or by consulting a third reviewer.

### 2.7 Data extraction, analysis, and presentation

Firstly, the articles included for full-text reading and data extraction were distributed equally among four reviewers. The first author developed a charting form in Excel (Microsoft, Seattle, WA, USA) for data extraction. In order to increase consistency and credibility, the charting form was discussed and adjusted by all reviewers after a pilot test involving the charting of four articles. The following data were extracted to describe the included articles: first author, publication year, country of origin, study objectives/aims, study design, participants, setting/rehabilitation sector, deliverers of the intervention, and a description of the tools and approaches used in the goal-setting process. Throughout the entire data extraction process, any uncertainty was discussed with a co-reviewer.

Secondly, inductive content analysis of the included studies was used to identify key elements related to goal-setting activities (31). To validate the analysis process, four reviewers (IR, MNM, PL, and HKK) independently coded two randomly selected articles and discussed the emerging codes. Then, the first author (IR) coded all the included articles. To refine and validate the coding, another reviewer (MNM) independently coded six randomly selected articles. Any discrepancies were discussed among the two reviewers or with a third reviewer (HKK). The qualitative data analysis software program NVivo (version 14.23.3; Lumivero, Denver, CO, USA) was used. The inductive codes were grouped into categories related to the goal-setting activities, and all categories were collated in Excel (version 2408; Microsoft, Seattle, WA, USA).

Finally, based on Wade (16), we defined the underlying domains in goal-setting processes to be (1) person-centeredness, (2) collaboration between healthcare professionals and patients/carers, (3) coordination across disciplines and sectors, (4) monitoring, and (5) evaluation. These domains were used to discuss the results of the included articles to generate an overview and identify gaps and overlaps in the goal-setting processes. The domains were based upon definitions of stroke rehabilitation and goal-setting, which indicate that goals are expected to facilitate the patient's autonomy, motivate the patient, ensure the team focuses on the same goals, ensure important actions are not overlooked, ensure monitoring of changes to end ineffective interventions, improve coordination between health professionals, patients, and carers, and support patients in acknowledging and accepting the consequences of a stroke (11, 16, 32).

## 3 Results

**Figure 1:**
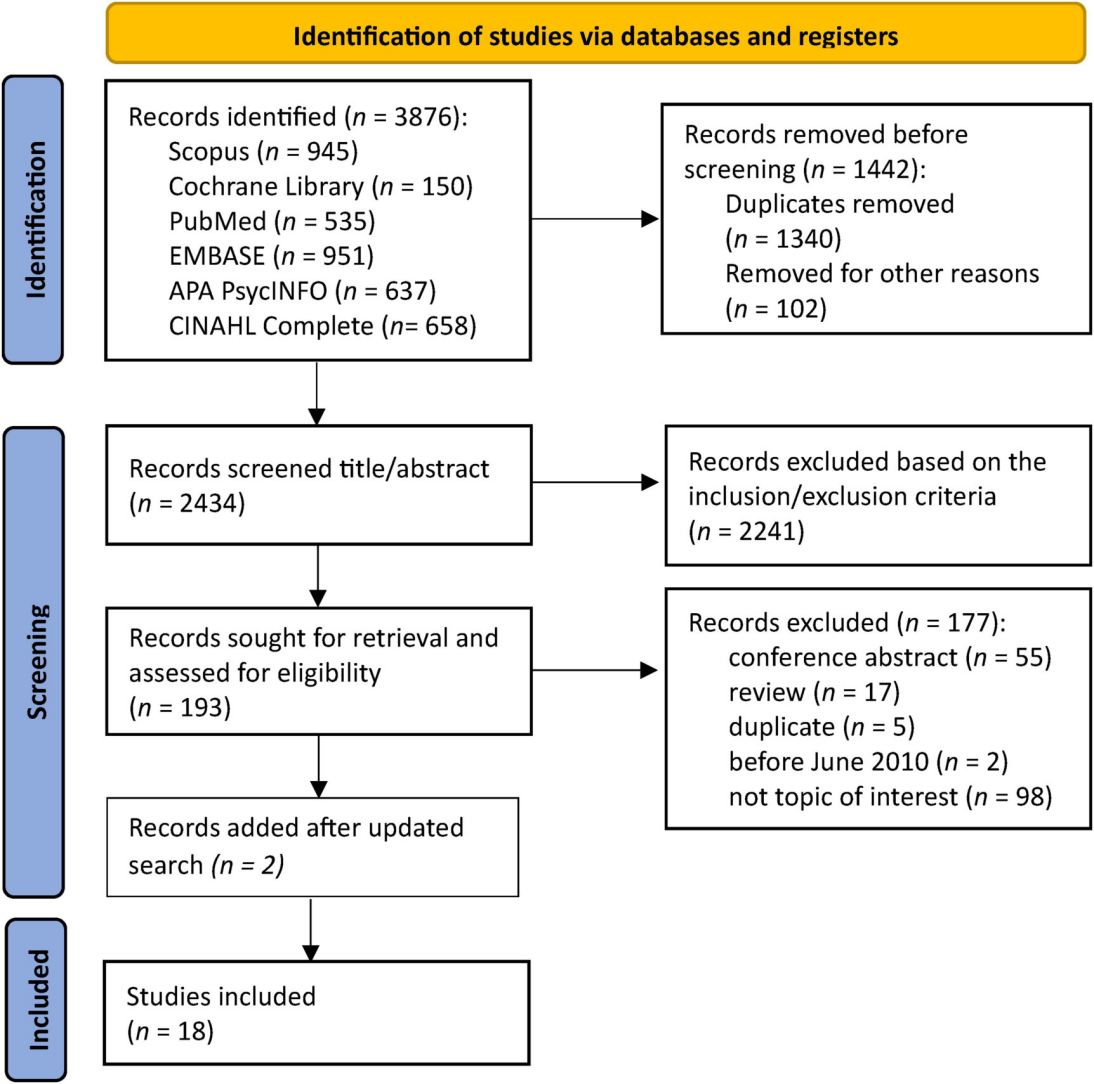
PRISMA flowchart of study inclusion and exclusion in the scoping review.

The initial search identified 3876 articles (Figure 1). The search strategy for each database is described in Supplement Material S1. After removing duplicates and excluding articles based on the inclusion and exclusion criteria, the full text of 193 articles was reviewed. Among the 177 articles that were subsequently excluded, the main reasons for exclusion were the study not addressing the review topic (*n* = 98) or the article being a conference abstract (*n* = 55). Sixteen studies were included in the analysis. The second search in 2025 added two studies (21, 33) No additional studies were identified by manually searching the reference lists of reviews identified during the search. The included studies are described and summarized in detail in Supplementary Table S1.

### 3.1 Studies design

Eight of the included studies used a qualitative design (interviews and observations) (21, 34–40), seven used a mixed-methods design (41–47), one used a survey (33), one used a retrospective design (48), and one used a prospective study design (49).

### 3.2 Participants

Four of the included studies focused on clinicians (33, 34, 37, 45), seven on patients (21, 35, 40, 44, 46, 48, 49), and seven on both (36, 38, 39, 41–43, 47). Physiotherapists, occupational therapists, and medical doctors, as well as dieticians, speech therapists, psychologists, nurses, and chaplains, were involved in the goal-setting processes. One study included persons with stroke and sclerosis; however, the authors described no notable differences between diagnosis groups when evaluating a goal-setting tool (36).

### 3.3 Settings

The setting varied across studies, including community rehabilitation centers (21, 37, 38, 46), inpatient and outpatient clinics (35, 36, 40, 42–44, 47, 48), and hospital units specialized in neurological (34, 43, 45, 47, 49), geriatric (39), home-based (40, 41) rehabilitation or a combination of these (33).

### 3.4 Timepoints

Nine of the included studies described the timing of goal-setting, dividing the rehabilitation process into stages (36, 38–41, 44, 46, 48, 49). Overall, the goal-setting process could be divided into three stages: an early stage, where the goals are identified; the rehabilitation stage, where the goals are adjusted or not; and finally, the discharge stage.

### 3.5 Tools and approaches

The specific tools and approaches during the goal-setting process varied across studies. One study used a package with a 34-item menu aligned with a clinician procedure manual containing guidelines on common goals, SMART (Specific, Measurable, Attainable/Achievable, Relevant, and Timely) goals (50), evidence-based strategies, goal-setting recording templates, and a summary sheet for patients (47). Three studies (21, 33, 34) found that clinicians used non-specific strategies to set goals. Goals could be standardized or therapist-led. Kessler et al. observed introductory and goal-selection actions (35). The latter exhibited four different patterns of interactions leading towards goal endorsement: (1) goal presentation with immediate endorsement of the goal, (2) goal exploration then endorsement, (3) tentative goal presentation and goal drop, and (4) persistent goal proposal until endorsed. Littooij et al. examined a goal-setting process based on patients' global values and worldviews, which guided the direction for identifying rehabilitation goals (36). Plant et al. found that goal-setting was mainly therapist-led and focused on mobility and activities of daily living goals in an inpatient stroke rehabilitation setting. In community-based rehabilitation (43). Scobbie et al. reported that a four-stage theory-based goal-setting and action-planning framework was used to guide goal-setting practice: goal negotiation, goal-setting, action and coping planning, and appraisal and feedback. In a geriatric rehabilitation setting (38). Smit et al. reported that the Collaborative Functional Goal-setting method was used to structure the goal-setting process and to integrate the patient's personal rehabilitation goals into measurable, standardized functional goals: the Barthel Index (BI) and the Utrecht Scale for Evaluation of Rehabilitation (USER) (39). Finally, studies in the UK used data based on the Goal Attainment Scale (GAS)-Light in an inpatient tertiary specialist rehabilitation where the patient identified one to six personal goals, which were transformed into SMART goals and rated using the GAS at the end of the program (49, 51).

### 3.6 Procedures related to goal-setting

Using an inductive content analysis of the included articles, we identified elements related to goal-setting in the different phases of stroke rehabilitation. The following themes were identified: description of goal setting processes (types of goals, goal setting tools, time for goal-setting), collaboration therapist-patient (communication, clarifying goals, prioritizing goals), adjusting goals (identifying barriers/facilitators, breaking down goals), documentation (monitoring goals, measuring goals).

In the first stage, the main procedures were introducing goal-setting, identifying and negotiating goals, establishing baseline levels, and addressing patient insecurity in realizing goals. Some elements used at this stage were also covered later in the rehabilitation stage: how to prioritize goals, manage barriers, break down goals into smaller goals, and use SMART goals or action and coping planning strategies. Adjusting goals was related to upgrading or downgrading goals.

**Figure 2:**
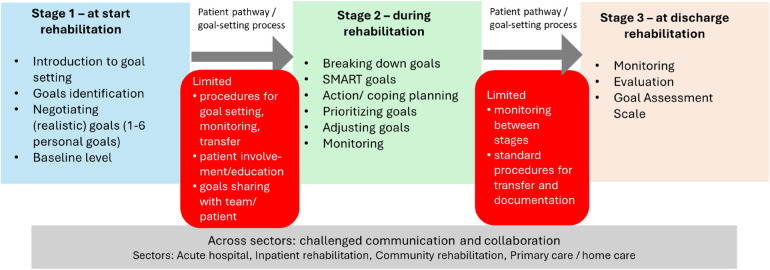
Stages of patient-centred goal setting in stroke rehabilitation, with key activities in each phase and points of breakdown in coordination across sectors. The arrows depict the progression from beginning rehabilitation through ongoing rehabilitation to discharge. The lower bar illustrates involvement of different care sectors throughout stroke rehabilitation. The red boxes highlight the transitions where communication and documentation of goals are often insufficient.

In the rehabilitation and discharge stages, different procedures for documenting goal-setting and monitoring set goals were described, such as the Goal Satisfaction Scale or clinical judgment (43, 48). Finally, in the discharge stage, evaluation procedures, such as the GAS, were described. Regardless of the stage, goal-setting could depend on the setting (systems, organizations, or environments), which influences the processes. Described as the balance of the clinician or patient/family having the leading role in the goal-setting process, collaboration was an element throughout the rehabilitation process. Different communication strategies in goal-setting processes were also described throughout the rehabilitation process. The rehabilitation process would lead to a transfer to another sector. This inter-sectorial transfer was generally poor or, mostly, not described in the included studies. This is illustrated in Figure 2.

## 4 Discussion

This scoping review identified a comprehensive picture of the existing research-based knowledge on goal-setting in stroke rehabilitation. To summarize and discuss the results, we aligned them with the five underlying domains in goal-setting processes described earlier in the method section: person-centeredness, collaboration between healthcare professionals and patients/carers, coordination across disciplines/sectors, monitoring, and evaluation. The alignment with the five domains in goal-setting processes generated an overview and enabled us to identify gaps and overlaps in the described goal-setting processes.

### 4.1 Patient-centeredness

Both patient-centeredness and person-centeredness include the following domains: 1) empathy, 2) respect, 3) engagement, 4) relationship, 5) communication, 6) shared decision-making, 7) holistic focus, 8) individualized focus, and 9) coordinated care. Though in some studies, person-centered care is more aiming towards a meaningful life and patient-centered care more towards a functional life (18). In this study, we did not distinguish between the two as our focus was the process in goal-setting.

In some studies, patient-centeredness was achieved by allowing patients to define goals without restrictions (35, 36, 38, 41, 48, 49). In other studies, the goals were predefined (47), limited to disability (43, 44, 49), or were a specific number (47). This finding aligns with the conclusion of a systematic review on stroke patients experiences with goal-setting that synthesized four findings (23): (1) patient-centered goal-setting is possible but often does not occur, (2) practitioners shape the context of goal-setting, (3) there is a need for an individualized approach to goal-setting, and (4) recovery is ongoing and unpredictable.

People with cognitive impairments are not specifically mentioned. However, regardless of the level of cognitive impairment, goal-setting is important to improve participation and mutual understanding of their goals. Clinicians should not distinguish between goal-setting strategies for patients with communication or cognitive impairments, as each patient has different needs. Therefore, clinicians should use a repertoire of strategies to best meet their needs (34). However, five domains seemed to be important for this large group of stroke patients: being flexible, creating trust in relations, enabling empowerment, communication skills for one-to-one interaction, and involving relatives (34).

### 4.2 Collaboration

The involvement of patients, clinicians, and interdisciplinary teams in goal-setting varied among the included studies, ranging from mainly patient-led, driven by collaboration between the patient and therapist (35), to mainly clinician-led (21, 39). In other studies, goals were defined by a multidisciplinary team (46). This variation was previously documented in a review that concluded that patients were often uncertain about their role in the goal-setting process and did not participate fully, whereas professionals seemed to be more positive about the level of collaboration with their patients in goal-setting. This review concluded that better collaboration and communication between professionals and patients were needed (20). A newer integrative review identified that therapeutic relationships were important for patient engagement in stroke rehabilitation (52). Stroke survivors can also have different views on goal-setting than health professionals. Therefore, communicating with patients to understand their views on goal-setting can enhance collaboration in the goal-setting process (53).

### 4.3 Coordination across disciplines/sectors

Most of the studies included did not describe coordination across sectors in detail. None of the included studies focused on cross-sectoral coordination and coherence. A recent review concluded that hospital-to-home transition is challenging the rehabilitation process, recommending protocols and guidelines to plan discharge and individual transitional care (54). This recommendation is supported by nine patients discharged after a stroke who needed greater communication and care coordination, emotional support, and fair access to services and support (55). A recent review identified aspects that help patients to self-manage after discharge to improve health outcomes and quality of life (56): pre-discharge assessment and planning; provision of continuous education and training; collaborative goal-setting; reinforcement and documentation of vital information; coordination of post-discharge care; provision of rehabilitation support and promoting community reintegration; provision of counseling support; and using clear communication, patient empowerment, and promoting self-efficacy. These aspects underscore the importance and complexity of coordination and communication across sectors. The (5) recommendations regarding discharge also stress the importance of information to all involved, including a health and social plan and standard procedures for discharge.

### 4.4 Monitoring and evaluation

Monitoring patients' rehabilitation is a dynamic process that requires continuous adjustment. Monitoring patients' outcome measures also impacts on the consideration of home discharge; the better the score, the greater the likelihood of discharge (57). Therefore, choosing how to monitor rehabilitation is of great importance.

In our scoping review, the time point for goal-setting varied from within the first 48 h, the first session, the first week/10 days, before the first team meeting, or throughout the program. Different tools and instruments were used to monitor or document the goals: the Canadian Occupational Performance Measure (COPM) (44), SMART (36, 43, 47, 49), GAS (47, 49), 10-m walk test and the Rivermead Mobility Index (41), goal-setting and action planning framework (38), Goal Satisfaction Score on an 11-point scale (48), a self-report measure of self-efficacy (38), and BI or the functional items of the USER (fUSER) (39). Different goal outcomes are used during the rehabilitation process. The International Classification of Functioning, Disability and Health (ICF) framework can assist in defining which domain could be focused upon in monitoring the goal-setting process by describing individual levels of functioning and disability, setting treatment goals, and identifying barriers and facilitators to individual functioning and health (58). Another component of monitoring could be motivation, which could be regularly assessed as the rehabilitation process can be long-lasting and prone to multiple changes. Frequent assessment of patients' motivation can help identify possible motivation decreases and, potentially, react appropriately by adapting therapeutic strategies (59).

Finally, it should be considered that the environmental setting and resources are important for goal-setting procedures, including who participates, the clinician's knowledge, physical locations, and the time allocated (60). However, how goal-setting is monitored in a hospital setting might not be transferable to a home-based setting.

### 4.5 Clinical implications of the findings

In summary, goal-setting processes in stroke rehabilitation practices are heterogeneous and not always supported by definitions of goal-setting. Our scoping review supports the need to implement the recommendations stated in 2023 by NICE for goal-setting in stroke rehabilitation as guidance to goal-setting processes. We recommend 1) the organization of processes to ensure that patients have meaningful and relevant goals that focus on activity and participation, both short- and long-term; 2) create settings (time and space) for timetabled and regular goal-setting meetings, involving the patient and, where appropriate, their carers; (3) educate patients by providing material that explains the goal-setting process and the information and support they need to make decisions and actively engage in goal-setting goals; (4) create templates to document agreed goals for stroke rehabilitation after each goal-setting meeting for both patients and team members; and (5) create processes to review patients' goals at regular intervals during their stroke rehabilitation (5).

Several tools are available to implement these recommendations. A recent review identified 55 different goal-setting tools in adult rehabilitation, with 55% used for goal selection and documentation, 27% for goal-setting and intervention delivery, and 16% for quality of goal-setting (61). Organizations, clinicians, and researchers should assess which set of goal-setting tools could be relevant for their rehabilitation processes. A study in Australia evaluated a goal-setting implementation package where five rehabilitation sites selected components of the goal-setting implementation package that they felt were feasible to implement (62). The evaluation of this study showed the vital importance of teamwork to successfully implementing goal-setting in clinical practice. Barriers for implementation were the lack of dedicated facilitation beyond the end of the implementation period. Facilitators to drive change within the rehabilitation team were the use of templates and prompts embedded in existing processes and regular audits and feedback (60).

Our study had inherent limitations due to its design, as a scoping review aims to provide broad information on the topic. However, the breadth of the research and the topic's clinical importance and variety justify using this design. To ensure that we addressed challenges in goal-setting processes linked to comparable practices, we limited our search to Western industrialized countries, which enabled us to draw clinically relevant assumptions. Another limitation of this scoping review was its exclusion of grey literature (i.e., conference papers or research theses), which may have overlooked relevant information on the topic. However, we screened the excluded conference abstracts and evaluated that these did not add new knowledge to this scoping review.

## 5 Conclusions

Patient-centered goal-setting in stroke rehabilitation is practiced with appreciable variability in high-income countries. No general procedure exists to ensure that goal-setting is patient-centered, with both therapist- and team-led goal-setting observed. Goal-setting activities occur more frequently in the first phase of stroke rehabilitation. Ongoing monitoring and evaluation tools are described to a lesser extent. Procedures regarding cross-sectoral rehabilitation are seldom described despite the importance of coordinated and coherent rehabilitation across sectors.
