# Supplementary material for: Patient-centered goal-setting in stroke rehabilitation: a scoping review
Source: Front Rehabil Sci. 2026 Mar 17;7:1744900. doi: 10.3389/fresc.2026.1744900 (PMC13036118; doi:10.3389/fresc.2026.1744900)
Supplement: Supplementary file 1 [file Table1.docx]

## Tables

| **Table 1. Details of the included articles.** | | | | | |
| --- | --- | --- | --- | --- | --- |
|  | | | | | |
|  | | | | | |
| **First author, year, country** | **Objectives/aims** | **Study design** | **Participant or patient target group** | **Setting or rehabilitation sector/Delivering professionals** | **Tool and/or approaches of the goal-setting process** |
| **Baker, 2022, Australia** | To review goal-setting practices across the rehabilitation continuum within varied case mix services. | A multisite qualitative case study with medical record audits and interviews with patients focusing on the process, action planning, and review of goals, as well as the type, specificity, and patient-centeredness of the set goals. | Medical record audits of 132 patients and 64 interviews; mean age of 66.09 ± 13.30 years; 39% recovering from a stroke, 19% recovering post-medical illness, and 13% recovering after orthopaedic surgery. | Inpatient or community rehabilitation settings. / Unclear who delivered. | The goals were set at admission, by rotating staff members, or by one designated staff member. The goals were also set by the rehabilitation teams without the patient present. Goals (*n* = 1120) were often poorly defined, focused on short-term physical functioning, and predominantly set by individual disciplines with the patient rather than an interdisciplinary approach. Patients were not consistently given action plans to pursue goals (*n* = 59, 18%). Few patients reported receiving copies of their rehabilitation goals (*n* = 16, 25%). |
| **Barnden, 2022, Australia** | To develop a goal-setting discharge package with patients recovering from a stroke, their support individuals, experts, and clinicians, and field test this package with clinicians. | A mixed methods study using participatory design to develop, train clinicians, field test, and evaluate a goal-setting package. | Leading academics, clinicians working in stroke, and patients recovering from a stroke and/or their support individuals. | One acute stroke unit and one inpatient stroke rehabilitation unit within a single Australian public health service. Nurses, occupational therapists, speech therapists, and dieticians. | The standardized goal-setting package included a 34-item menu aligned to a clinician procedure manual containing guideline summaries, common goals, SMART goal-metrics, evidence-based strategies and worked examples, goal-setting recording templates based on GAS methodology, a summary sheet for patients, and a comprehensive goal-setting training package. The “menu” was provided to all patients recovering from a stroke, and patients led the selection of goal priority areas using agreed-upon strategies. They could translate a broad goal area into a very specific SMART goal. All patients were provided with a copy of the patient summary sheet. |
| **Dörfler, 2020, Austria** | To explore strategies used in rehabilitation to involve stroke survivors with communication and/or cognitive impairment in patient-centered goal-setting. | Semi-structured in-depth interviews with stroke rehabilitation professionals and thematic analysis to describe self-reported practice-based strategies from health professionals’ clinical experience. | Patients with communication and/or cognitive impairment after a stroke. | Acute (*n* = 1), inpatient (*n* = 8), and outpatient (*n* = 3) rehabilitation and education (*n* = 3) settings. / 11 stroke rehabilitation professionals: occupational therapists (*n* = 5), therapists, physiotherapists (*n* = 1), speech and language therapists (*n* = 3), and psychologists (*n* = 2) with two years of clinical experience. | Twenty-one aspects of person-centered goal-setting were described and grouped according to five themes: flexibility, trusting relationships, enabling empowerment, techniques for one-to-one interaction, and involving relatives. |
| **Kersten, 2015, New Zealand** | To test the feasibility and acceptability of an implementation intention strategy (if-then plans) to bridge the goal intention-action gap in rehabilitation with patients with neurological conditions experiencing mobility difficulties from the perspectives of patients and therapists. | A mixed methods study in which patients were randomized to experimental (goal-setting augmented by if-then plans) and control (goal-setting only) groups to set up three mobility-related goals with a physiotherapist. Focus groups and interviews with patients and therapists. | 20 patients, 10 with multiple sclerosis and 10 recovering from a stroke. | Patients’ homes. / Four physiotherapists. | Session 1 was conducted face-to-face in the participants’ homes. Participants set one to three exercise or activity goals for their self-managed, mobility-related rehabilitation with the therapist. The therapist ensured that these were formulated using best practice guidance and that they were specific and measurable. The goals were recorded on a purposely developed data sheet; one copy was held by the participant, and one by the physiotherapist. The physiotherapist made a follow-up call three weeks later. The physiotherapist mailed a record of the outcomes of this session to the participants for their records. The experimental intervention received the same best practice goal-setting. After developing goals with the participants, facilitators and barriers to performing each activity or exercise goal were discussed to target “real” or “meaningful” implementation intentions. Physiotherapists then supported participants in formulating if-then plans for each goal. |
| **Kessler, 2019, Canada** | To determine what might facilitate or impede the formation of patient-centered goals in a context highly supportive of patient-centered goal-setting. | Conversational analysis to examine goal-setting conversations. Twelve goal-setting sessions were purposively selected, transcribed, and analyzed. | 12 patients recovering from a stroke (mean time since stroke: 60.6 weeks): 7 males (mean age: 70.85 [37–94] years), and 5 females (mean age: 67.6 [49–85] years). | Inpatient rehabilitation or outpatient stroke rehabilitation. / Two occupational therapists with seven years of clinical experience that included working with patients who had experienced a stroke. | Two types of interactions were observed: (1) Introductory actions set the context for goal-setting and involved sharing information and seeking clarification related to goal requirements and patients’ occupational performance competencies; (2) Goal selection actions using four patterns of interactions in which the goals were explored and then endorsed or dropped: (i) goal presentation with immediate endorsement of the goal, (ii) goal exploration then endorsement, (iii) tentative goal presentation and goal drop, and (iv) persistent goal proposal until endorsed. |
| **Laver, 2010, Australia** | To describe the participants’ readiness and ability to set goals over time. | Qualitative study using semi-structured interviews with patients at three different time points. | 15 stroke survivors aged 18–70 years. | Acute care, subacute rehabilitation, and six months later at home. / Medical practitioners, occupational therapists, physiotherapists, speech pathologists, and nurses. | No formal goal-setting tools were used. Participants had trouble setting goals early after the stroke due to limited understanding of goal terminology, wanting to “get back to normal,” differences in readiness to set goals, and poor knowledge about stroke and stroke recovery. |
| **Levack, 2011, New Zealand** | To examine the application of goal-setting in inpatient stroke rehabilitation. | Constructivist grounded-theory analyses of interviews, recorded clinical sessions, team meetings, participant observations, and clinical documentation. | 9 patients and 7 family members. | Two inpatient stroke rehabilitation / Twenty-eight health professionals: 6 physicians, 11 registered nurses, 4 physiotherapists, 3 occupational therapists, 2 social workers, 1 speech-language therapist, and 1 cultural advisor. | Both rehabilitation units used a documented interdisciplinary team plan for each patient, established protocols for gathering information about the views held by patients and families regarding their aims for rehabilitation, and had nominated staff members to collect this information using a structured questionnaire. In addition to patient and family goals, both units also documented shared team goals, which were derived from the patient and family goals. Some goals (characterized by short timeframes, conservative estimation of outcomes, and physical function) were prioritized within the rehabilitation process over others. The involvement of patients and their families in goal-setting resulted in interactional dilemmas when their objectives, skills, and perceived capacity did not align with prioritized goals. |
| **Littooij, 2022, Netherlands** | To evaluate the experience of patients and clinicians in working with a tool to help set goals that are personally meaningful to patients undergoing rehabilitation. | Individual, semi-structured interviews and focus group interviews with patients and clinicians, respectively. | 7 males and 11 females aged 23–67 years (mean: 46); 8 were recovering from their first stroke, and 10 had multiple sclerosis. | A university medical center and a rehabilitation center. / Eight rehabilitation physicians, three chaplains, and 27 other rehabilitation team members (eight physical therapists, seven occupational therapists, two speech therapists, one sports therapist, six psychologists, and three social workers). | The client, chaplain, and rehabilitation physician (1) explored the patient’s global meaning (relationships, core values, worldview, identity, and inner posture) and (2) identified the meaningful overall rehabilitation goal. The other rehabilitation team members and the patient (3) set specific rehabilitation goals that served to achieve the meaningful overall rehabilitation goal. The specific goals were recorded in the patient’s file. The specific goals could be adjusted or revised during the rehabilitation trajectory, depending on the course of the rehabilitation process. |
| **Lloyd, 2014, UK** | To investigate physiotherapists’ perceptions about their experiences of collaborative goal-setting with patients in the sub-acute post-stroke stages in a hospital setting. | Semi-structured interviews with physiotherapists. The transcripts were coded and analyzed using the constant comparative method of grounded theory. | Patients in the subacute post-stroke stages in the hospital setting. | National Health Service hospitals. / Nine registered physiotherapists recruited from three stroke units, aged 20–50 years, with one month to 16 years of experience in stroke rehabilitation. | Themes on physiotherapists’ perceptions about involving patients in goal-setting: (1) “Coming to terms with stroke”: the individual patient journey, health factors, personal factors, environmental factors, passing of time; (2) “The evolution of goalsetting skill”: individual physiotherapist journey, changing focus, learned and innate component; (3) “Finding a balance”: managing expectations, negotiating interactions, influence of workplace culture; (4) “You are the expert”: balance of power and control. |
| **Notkin, 2025, Denmark** | To explore patients’ experiences with rehabilitation plans in relation to their transition from hospital admission to the post discharge period | Semi-structured interviews with patients in municipality rehabilitation, post discharge from hospital. | Patients discharged from hospital after a hemorrhagic or ischemic stroke. | Municipalities in Denmark / Physiotherapists | Not reported specifically. Lack of patient involvement and patients are challenged in defining goals. Goals mostly standardized goals and/or caretaker- or therapists led |
| **Parsons, 2018, UK** | To investigate stroke rehabilitation clinicians’ perceptions of the patient as an active partner in setting goals within stroke rehabilitation and factors that influence patient engagement. | Semi-structured interviews with clinicians, with the transcripts subjected to general inductive analysis. | Patients recovering from a stroke. | An acute stroke ward and two community-based rehabilitation teams. / Twenty clinicians (seven occupational therapists, five physiotherapists, and eight speech therapists) with a mean age of 36.1 years and a mean time since qualification of 9.6 years. | There was universal recognition among the clinicians that goals had to be meaningful to the patient to maximize active engagement in rehabilitation. The complexity of identifying goals collaboratively with patients after a stroke was highlighted, and the interviewees were aware that formal tools such as the Canadian Occupational Performance Measure (COPM) or Mood Disorder Scales could facilitate discussion and identification of issues from the patient’s perspective. Activity breakdown (where longer-term goals are broken down into smaller, shorter-term goals) was a key strategy for communicating the rationale for treatment options to patients and linking them to the patient’s identified wishes. |
| **Plant, 2018, UK** | To explore how goal-setting is practiced during inpatient stroke rehabilitation; the nature and focus of the set goals; who was involved; and how goals are set, monitored, progressed, and integrated with other aspects of rehabilitation processes. | Mixed-methods: an online survey of stroke rehabilitation teams regarding their goal-setting practice and views, as well as documentary analysis of the clinical records related to goal-setting. | 49 adult patients recovering from a stroke receiving rehabilitation (mean time since stroke: 37 ± 44.4 days; mean BI: 7.8 ± 6.3). | Five National Health Services inpatient stroke units. / Six physiotherapists, five occupational therapists, one speech and language therapist, and one dietitian. Five (39%) worked in an acute stroke unit, two (15%) in a rehabilitation unit, and six (46%) in a combined acute and rehabilitation unit. Most were specialists or highly specialized therapists. Most had been using goal-setting for over 3 years. | Goal-setting was included in weekly multidisciplinary team meetings, which also covered assessment, monitoring progress, and action/treatment plans. None used a specific goal-setting method or tool. All units identified the patients’ goals in the first meeting, typically within the first week after the stroke or admission and noted the date the goal was set and achieved. Two units phrased the goals in terms of the profession setting them (professionals’ goals, rather than the patients’), one used predefined areas of function to focus on the goals (transfers, eating, or dressing), and two did not structure the goals. One unit specified a timescale for completion of the goal or how completion would be judged/assessed. Overall, 143 (41%) of documented goals met the SMART criteria. One unit never used the SMART format; the other units ranged between 21% and 82% of documented goals. Observation was the most frequent method used to assess whether a goal had been achieved (*n* = 21, 6%). Standardized outcomes were stated in 20 (6%) goals. The connection between the goal and treatment plans was usually vague and amounted to suggestions of the type of treatment modality that staff might employ. |
| **Rice, 2017, Canada** | To categorize goals that patients wanted to achieve while in therapy, to classify patient goals within the ICF framework, and to compare patients’ satisfaction scores at admission and discharge between the ICF categories of goals. | Retrospective chart review of patients attending an outpatient stroke rehabilitation program. | 286 stroke patients (58% male, mean time since onset: 6.0 months, mean age: 63.0 years); enrolled and attended at least one therapy session (i.e., physiotherapy, occupational therapy, or speech-language therapy) and after the initial intake appointment, the patient set at least one goal during enrollment. | Hospital-based, time-limited outpatient rehabilitation. / Physiotherapist, occupational therapist, or speech/language pathologist. | Patients attend an intake appointment that involves a general screening for eligibility and completing preliminary assessments with a physiotherapist, occupational therapist, or speech/language pathologist to consider the severity of their deficits. Therapy was not administered during the intake appointment. After the intake appointment during preliminary assessments, patients stated the goals (up to 10) they wanted to achieve during therapy, and an occupational therapist or physiotherapist recorded these goals in order that the patient stated them on a standardized form. While all goals were set by the patient, during rehabilitation, if a therapist believed that a patient’s goal cannot be safely achieved during the time frame of rehabilitation, they worked collaboratively with the patient to break down the goal into smaller, more achievable components and then noted this on the goal satisfaction form. At the time of goal-setting, patients rated their current satisfaction with their ability to perform each goal on an 11-point scale from 0 (not satisfied) to 10 (most satisfied). |
| **Scobbie, 2013, UK** | To investigate the implementation, acceptability, and perceived benefits of a goal-setting and action planning framework (G-AP), and to explore one community rehabilitation team’s experience of implementing it with patients recovering from a stroke. | In-depth interviews with patients and clinicians, with transcripts thematically analyzed. Case notes were analyzed descriptively. | Patients recovering from a stroke: 5 males and 3 females aged 29–78 years. | Community rehabilitation team (ReACH team). / Two occupational therapists, two physiotherapists, one dietitian, one nurse, and two speech and language therapists. | Four stages of G-AP: In the *goal negotiation* stage, patients consider their current situation and identify the main problem(s) they want to address. In the *goal-setting* stage, the identified problem is refined into a specific, challenging rehabilitation goal agreed upon by both health professionals and patients. *Action plans* detail what the patient must do (in sequential steps) to meet the goal, and *coping plans* detail strategies to be activated if barriers hinder action plan attainment. A self-report measure of self-efficacy is included in the planning stage to assess patients’ confidence to complete set plans; a lack of confidence (score of <7) suggests the plan should be modified to optimize the likelihood of the patient following through with it. Finally, the *appraisal and feedback* stage prompts a progress review and feedback from the health professional to the patient. |
| **Smit, 2018, Netherlands** | To explore the feasibility of collaborative functional goal-setting by exploring the views and experiences of both patients and professionals with the intervention during inpatient geriatric rehabilitation. | Interviews with patients and professionals. | Patients recovering from a stroke: 5 males and 3 females aged 63–87 years. | Inpatient geriatric rehabilitation wards. / Two elderly care physicians and one nurse practitioner. | (1) The Barthel Index (BI) or Utrecht Scale for Evaluation of Rehabilitation (fUSER) is completed within 48 hours of admission. (2) The test scores are presented to the team. (3) Attainable functional goals are set at the multidisciplinary team meeting (MDM), which are target scores on corresponding items of the BI or fUSER. (4) The MDM is followed by a goal-setting meeting with the patient and the physician or nurse practitioner, where the patient is invited to set their personal functional goals. (5) Shared decision-making is ensured in defining the patient’s goals by: (a) Elicit the patient’s views on the degree of involvement in decision-making; (b) The patient and physician jointly set functional goals based on consensus; (c) The physician translates these goals into target scores on the corresponding items of BI or fUSER. (6) Before every biweekly MDM, a new functional assessment is conducted. (7) During the MDMs, the functional goals and assessment target scores are reviewed: (a) No adjustment; (b) A higher target score proposed to the patient; (c) A lower target score proposed to the patient. (8) The patient is informed about the status of the functional rehabilitation goals, and the proposed alterations in functional goals are agreed upon. (9) When all potentially adjusted goals are met, the patient can be discharged from the rehabilitation program. |
| **Turner-Stokes, 2015, UK** | To examine the relationship between patient/family engagement in goal planning, satisfaction with the goal-setting process, and the associated goal attainment and functional gains during rehabilitation. | A prospective cohort analysis of consecutively completed episodes for patients discharged over one year. | 83 adults with neurological disabilities (mean age: 42.8 ± 15.0 years); program length: 98 ± 47 days; male-to-female ratio: 69:31; diagnosis: brain injury (*n* = 75, 90%), spinal cord injury (*n* = 5, 6%), other neurological conditions (*n* = 3, 4%). | Inpatient tertiary specialist rehabilitation service. / Team members. | One to six key “personal goal-objectives” for the program are identified and agreed on by the patient (and/or their family carer) and their treating team. Each goal-objective is rated for importance by the patient and/or their family carer on a scale from 1 (fairly) to 3 (very important). A SMART goal statement is drawn up to describe the expected level of achievement for each goal objective. Short-term staged goals towards the identified key goal objectives are set and reviewed at fortnightly intervals throughout the program to determine whether the patient is on track. Goal attainment is rated against the key SMART goal objectives with the patient and/or their family carer in a review meeting during the final week of the program. The attainment of each goal objective is rated on a six-point verbal scale and converted to a five-point numerical scale (from −2 to +2). |
| **Yosef, 2019, Israel** | To explore the feasibility, acceptability, and preliminary efficacy of a metacognitive occupation-based intervention in a telerehabilitation format with adults and older adults in the chronic phase after acquired brain injury (ABI). | A quasi-experimental pilot study with participants completing a tele-intervention. | 5 community-dwelling participants (aged 65–72 years), 6–10 months post-ABI, with scores of 2–4 on the modified Rankin scale and without dementia (4 patients had experienced a stroke). | Community-based day rehabilitation. / Occupational therapist. | The intervention program included up to 15 Cognitive Orientation to Daily Occupational Performance (CO-OP) sessions, 1–2 times weekly (∼45 min per session), delivered via video conferencing using Skype over three months. Each participant identified five functional goals during the baseline assessment using the COPM, of which three were the focus of the intervention during the intervention sessions (untrained goals). At the first intervention session, the occupational therapist and participant reviewed and re-discussed the goals, and the occupational therapist taught the participant the global problem-solving strategy (Goal-Plan-Do-Check). In the following sessions, the occupational therapist guided the participant in using this strategy to help them discover their performance problems and potential task-specific strategies to improve their performance and enable goal attainment. Rather than providing the participant with the solutions, the occupational therapist facilitated this process with questions and feedback. Therefore, the participants performed some of the activities during sessions if possible, and the occupational therapist observed the actual performance in the participant’s natural environment via video conferencing. |
| **Vieira, 2025, Portugal** | To explore attitudes and practices toward goal-setting in stroke rehabilitation among Portuguese physiotherapists | An online cross-sectional  exploratory survey was conducted, collecting data on sociodemographic and service profiles, patient-centeredness,  attitudes and practices of goal-setting. | Patients in different phases of rehabilitation in Portugal | Inpatient unit, hospital, clinic, health centre, at home.  Physiotherapists  in Portugal, treating at least one stroke survivor/ month in any setting, phase, or condition. | 51.6 % did not use a specific instrument to guide goal-setting, almost 50 % stated the International Classification for Functioning (ICF) was never or rarely used as a guide for goal-setting. About 4 %  Used the Goal Attainment Scale, or Canadian Occupational Performance Measure. |
